# Does Celiac Disease Influence Survival in Sepsis? A Nationwide Longitudinal Study

**DOI:** 10.1371/journal.pone.0154663

**Published:** 2016-04-28

**Authors:** Anna Röckert Tjernberg, Jonas Bonnedahl, Jonas F. Ludvigsson

**Affiliations:** 1 Department of Pediatrics, Kalmar County Hospital, Kalmar, Sweden; 2 School of Health and Medical Sciences, Örebro University, Örebro, Sweden; 3 Department of Infectious Diseases, Kalmar County Hospital, Kalmar, Sweden; 4 Zoonotic Ecology and Epidemiology, Faculty of Health and Life Sciences, Linnaeus University, Kalmar, Sweden; 5 Department of Medical Epidemiology and Biostatistics, Karolinska Institutet, Stockholm, Sweden; 6 Department of Pediatrics, Örebro University Hospital, Örebro, Sweden; Tulane University, UNITED STATES

## Abstract

**Background:**

Individuals with celiac disease (CD) are at increased risk of sepsis. The aim of this study was to examine whether CD influences survival in sepsis of bacterial origin.

**Methods:**

Nationwide longitudinal registry-based study. Through data on small intestinal biopsies from Sweden’s 28 pathology departments, we identified 29,096 individuals with CD (villous atrophy, Marsh stage III). Each individual with CD was matched with five population-based controls. Among these, 5,470 had a record of sepsis according to the Swedish Patient Register (1,432 celiac individuals and 4,038 controls). Finally we retrieved data on mortality in sepsis patients through the Swedish Cause of Death Registry.

**Results:**

CD was associated with a 19% increase in overall mortality after sepsis (95% confidence interval (CI) = 1.09–1.29), with the highest relative risk occurring in children (adjusted hazard ratio (aHR) = 1.62; 95%CI = 0.67–3.91). However, aHR for death from sepsis was lower (aHR = 1.10) and failed to reach statistical significance (95%CI = 0.72–1.69). CD did not influence survival within 28 days after sepsis (aHR = 0.98; 95%CI = 0.80–1.19).

**Conclusions:**

Although individuals with CD seem to be at an increased risk of overall death after sepsis, that excess risk does not differ from the general excess mortality previously seen in celiac patients in Sweden. CD as such does not seem to influence short-term or sepsis-specific survival in individuals with sepsis and therefore is not an independent risk factor for poor prognosis in sepsis.

## Introduction

Celiac disease (CD) is a chronic immune-mediated disorder triggered by the ingestion of gluten in genetically susceptible individuals [1]. It occurs in approximately 1% of the western population and treatment consists of a life-long gluten-free diet [2]. CD has been associated with excess morbidity [3] as well as mortality [4–6]. In addition to an increased risk for malignancies [3] and different autoimmune diseases [7], individuals with CD are at an increased risk of severe infections [8] including tuberculosis [9], influenza [10] and pneumococcal infections [11]. In 2008 we reported an elevated risk for sepsis in patients with CD compared with a group of inpatient reference individuals (hazard ratio (HR) = 1.6; 95% confidence interval (CI) = 1.2–1.9) [12]. These findings supported earlier reports of an excess mortality from sepsis in CD [13].

Sepsis, which is characterized by a systemic inflammatory response with elevated or reduced body temperature, tachycardia, tachypnea and affected white blood cell count, is the body’s sometimes overwhelming reaction to an invasive infection. When organ dysfunction is added to these parameters, the condition is defined as severe sepsis [14, 15]. Incidence data of this life-threatening infection vary but studies report incidence numbers between 50–300 per 100,000 person-years, with a mortality ranging from 10–20% or even higher in severe sepsis and septic shock [14, 16].

Several CD-related factors may predispose to severe infections, including sepsis. One such mechanism is hyposplenism [17] although the susceptibility to infectious diseases has also been attributed to the altered intestinal permeability seen in CD [18]. The incidence of sepsis seems to increase and considering the high mortality [16] it is important to identify risk factors affecting its prognosis. The outcome in sepsis is related to several determinants including different underlying conditions [19, 20]. While a few studies have evaluated the risk of death in infectious diseases in CD [4, 13] we are not aware of any study exclusively examining whether CD affects the prognosis in patients with sepsis.

The aim of this population-based study was therefore to examine whether CD influences survival in sepsis of bacterial origin. We hypothesised that individuals with sepsis and concomitant CD would have a poorer survival than individuals without CD.

## Materials and Methods

### Study participants

During 2006–2008 data on small intestinal biopsy reports were collected from all of Sweden’s pathology departments (n = 28). The biopsies had been performed in 1969–2008 and data were retrieved by local IT personnel and included information on personal identity number [21], date of biopsy, topography (duodenum or jejunum) and morphology in compliance with the Swedish SnoMed classification codes (see S1 file), a classification used by all 28 departments. Details on this data collection process have been published previously [22]. CD was defined has having villous atrophy (VA, equivalent to Marsh stage III). We did not require positive serology for the CD diagnosis; however, a previous validation has shown that in a random sample of study participants with VA some 88% were positive for CD serology at time of biopsy [22], a proportion consistent with data from other countries [23]

After removal of duplicates and erroneous data we identified 29,096 individuals with VA (identical to the dataset used in our previous study on CD and mortality [6]). Each individual was thereafter matched with five controls from the Swedish Total Population Register (n = 144,522). Matching criteria included sex, age, county and year of biopsy.

### Sepsis

Through relevant international classifications of disease (ICD) codes (see S2 file), we were able to retrieve data on sepsis of bacterial origin from the Swedish Patient Register for all our study participants. The Patient Register started in 1964 and becoming nationwide in 1987 [24]. Initially, this register only contained inpatient care, but since 2001 the register includes also hospital-based outpatient care [24]. In this study sepsis was defined as having one of the ICD codes presented in the S2 file. Through linkage to the Patient Register we identified 5,470 individuals from the original data set who had received a sepsis diagnosis (CD: n = 1,432 (4.9%) and controls: n = 4,038 (2.8%)).

### Mortality

Data on death date and causes of death were retrieved from the Swedish Total Population Register and the Swedish Cause of Death Register, respectively.

### Demographic data

Data on socioeconomic status, educational level and country of birth were obtained from Statistics Sweden (the Swedish government agency responsible for producing official statistics). Socioeconomic status and education were divided a priori (for a detailed description, see Olén et al [25]).

### Statistics

The risks of overall death and death from sepsis were estimated using Cox regression. Because the original study enrolled individuals at date of biopsy and matched controls that were alive at the corresponding date, we chose a model that allowed for staggered entry. Follow-up ended at death, emigration or end of the study period (Dec 31, 2009) whichever occurred first. HRs were adjusted for sex, age at diagnosis of sepsis, subtype of sepsis, calendar period at sepsis, socioeconomic status, level of education and country of birth. In a separate analysis we adjusted for type 1 diabetes mellitus and autoimmune thyroiditis. Comorbidities were identified from the Patient Register (see S3 file for relevant ICD codes). Individuals with missing data on socioeconomic status and educational level were fitted into a separate category. A priori sub-analyses included stratification for age at diagnosis of sepsis (0–19, 20–39, 40–59, 60–79, 80- years), calendar period (1969–1989, 1990–1999, 2000–2009) and sepsis according to bacterial origin (streptococcal (excluding pneumococcal), pneumococcal, Gram-negative, staphylococcal and non-specified sepsis; all defined according to relevant ICD codes). A separate analysis was also done to examine whether risk estimates changed if we restricted the study population to include only individuals who received a sepsis diagnosis after the CD diagnosis.

We used SPSS 22 (SPSS, Inc. Chicago, IL, USA) and the R survival package for all analyses. HRs with a 95% confidence interval (CI) not including one and p < 0.05 were considered significant.

### Ethics

This study was approved by the Ethics Review board of Stockholm, Sweden. Because data were strictly register-based, individual consent was not required.

## Results

All participants in this study had a diagnosis of sepsis at some stage during life. Most study participants were diagnosed with sepsis at an older age (about 68% were 60 years or above) and the majority were diagnosed in the last calendar period (2000–2009). Approximately 48% of the study participants were females. Further information on characteristics of the study participants are presented in Table 1. Distribution of sepsis according to bacterial origin in the CD group versus the controls is summarised in Table 2.

**Table 1:** Characteristics of individuals with sepsis.

Characteristics	Controls n (%)	Celiac disease n (%)
Total	4038 (100)	1432 (100)
Sex		
Male	2149 (53.2)	697 (48.7)
Female	1889 (46.8)	735 (51.3)
Age^a^ (years)		
0–19	274 (6.8)	109 (7.6)
20–39	260 (6.4)	164 (11.5)
40–59	615 (15.2)	317 (22.1)
60–79	1845 (45.7)	655 (45.7)
80-	1044 (25.9)	187 (13.1)
Calendar year^a^		
1969–1989	435 (10.8)	165 (18.3)
1990–1999	1117 (27.7)	486 (45.0)
2000–2009	2486 (61.6)	781 (36.7)
Country of birth		
Nordic	3860 (95.6)	1377 (96.2)
Not Nordic	178 (4.4)	55 (3.8)

^a^ At time of sepsis diagnosis

**Table 2:** Distribution of sepsis according to bacterial origin.

Bacteria	Controls n (%)	Celiac disease n (%)
Streptococci (excl pneumococci)	212 (5.3)	77 (5.4)
Pneumococci	280 (6.9)	105 (7.3)
Meningococci	64 (1.6)	19 (1.3)
H. Influenzae	6 (0.1)	5 (0.3)
Gram-negative bacteriae	1061 (26.3)	348 (24.3)
Anaerobic bacteriae	50 (1.2)	14 (1.0)
Staphylococci	505 (12.5)	225 (15.7)
Unspecified	1860 (46.1)	639 (44.6)

### All-cause mortality

During follow-up, 3,155 individuals died (841 celiac individuals and 2,314 controls). CD was associated with an increased risk of death from any cause after sepsis (adjusted hazard ratio (aHR) = 1.19; 95%CI = 1.09–1.29). The highest mortality rates was seen in children with CD although the risk estimate did not attain statistical significance (HR = 1.67; 95%CI = 0.67–3.91). Nor was the separate risk increase for adults (≥20 years) statistically significant (HR = 1.03; 95%CI = 0.95–1.12). Having a sepsis diagnosis early in the study period (1969–1989) was associated with the highest relative risk of death (aHR = 1.76; 95%CI = 1.38–2.26). When analysing subtypes of sepsis (i.e. stratified after bacterial origin) we noted an increased overall mortality among individuals with CD following streptococcal, pneumococcal and Gram-negative sepsis. (Table 3)

**Table 3:** Mortality overall and according to bacterial origin^a^ of sepsis.

	All-cause mortality		Mortality from sepsis^b^	
	Number of deaths in CD-group; observed versus expected	aHR (95%CI)	Number of deaths in CD-group; observed versus expected	aHR (95%CI)
	841 vs 707	1.19 (1.09–1.29)	147 vs 134	1.10 (0.72–1.69)
Bacteria				
Streptococci	43 vs 26	1.66 (1.12–2.4)	1 vs 0.4	2.27 (0.15–47.9)
Pneumococci	53 vs 35	1.51 (1.06–2.16)	4 vs 2.5	1.57 (0.26–9.43)
Gram-negative	228 vs 174	1.31 (1.12–1.54)	4 vs 4.3	0.93 (0.32–2.73)
Staphylococci	131 vs 117	1.12 (0.90–1.39)	3 vs 8.8	0.34 (0.10–1.16)

^a^ Only in a minority of patients had a specific pathogen been specified. Table 3 lists the main pathogens recorded as cause of sepsis in our study.

^b^ Any sepsis

CD, celiac disease

aHR, adjusted Hazard ratio

Restricting our study participants to those with sepsis occurring after CD diagnosis or study entry (controls) we found a slightly lower risk for all-cause mortality (aHR = 1.12; 95%CI = 1.02–1.23). The HRs for children and adults separately were also lower but still not significant (children: 1.31; 95%CI = 0.43–4.0), adults: 0.98; 95%CI = 0.89–1.08). The first two calendar periods (1969–1989 and 1990–1999) in the study were associated with higher risk for death than the last calendar period, however, in this restricted population only the second period had a statistically significant risk increase. (Calendar period 1: aHR = 1.26; 95%CI = 0.71–2.06 and calendar period 2: aHR = 1.25; 95%CI = 1.05–1.48). The risk estimates changed only marginally when we restricted the study population to individuals with sepsis after CD diagnosis (data not shown).

### Mortality from sepsis

Of the 5470 individuals with a sepsis diagnosis, 147 had sepsis listed as their cause of death (CD: 33/1432 (4.9%); controls: 114/4038 (2.8%)). All deaths occurred in individuals diagnosed with CD in adulthood. Mortality from sepsis was higher in the CD group than in the non-CD group but the risk estimate failed to reach statistical significance (aHR = 1.10; 95%CI = 0.72–1.69). Risk estimates for death in sepsis according to the most common subtypes of sepsis are listed in Table 3. Because of insufficient power, we were unable to examine the risk for death according to subtype of sepsis. When broadening our definition “death from sepsis” to include not only cases where sepsis had been listed as the underlying cause of death but also cases where it was listed as contributing cause, we found an aHR of 1.15 (95% CI = 0.95–1.40).

Again, restricting our study participants to those who received a sepsis diagnosis after the CD diagnosis, the risk estimate decreased (aHR = 1.04; 95% = 0.67–1.62) and remained statistically non-significant. The relative risks presented in table 3 did not change substantially in this restricted study population

### Mortality within 28 days after sepsis diagnosis

Individuals with CD were at no increased risk of death within 28 days after sepsis compared with controls (aHR = 0.98; 95%CI = 0.80–1.19)

## Discussion

In this nationwide study that included nearly 5500 individuals with sepsis, patients with concomitant CD had an increased risk for overall death. This result is consistent with several previous studies, which similarly have found excess mortality in individuals with CD compared with the general population [4–6]. However, the 19% increase in risk for overall death was due to an excess mortality beyond the first month after sepsis. We did not find any increased mortality within 28 days after sepsis; nor did we find an increased mortality from sepsis specifically. Thus CD seems unlikely to influence survival in sepsis.

This study included patients from 1969 and the highest HR for overall death was observed among patients diagnosed with sepsis in the first part of the study period, between 1969 and 1989. This group was accordingly diagnosed with CD early in the study period when the use of serology was less common, the villous atrophy at diagnosis potentially more advanced than today and diagnosed cases were limited to symptomatic CD. Earlier research suggests that CD patients with malabsorption symptoms have a higher mortality than CD patients with minor symptoms [26]; such symptoms were more common in the first part of the study period [27]. Furthermore, research indicates that individuals with CD have lower levels of vitamin D than the general population [28] and vitamin D deficiency has also been linked to excess mortality [29]. Also, low levels of vitamin B have been linked to both untreated and treated CD [30]. In this study we did not have access to individual-based data on vitamin B-levels in the study participants. Although most research does not support a strong association between vitamin B-deficiencies and mortality in sepsis [31, 32], we cannot rule out that nutritional deficiencies may have influenced the sepsis mortality in some of our subanalyses. Even though the clinical presentation of CD has changed during the study period [27] we choose to include all celiac patients with sepsis to increase the statistical power. Restricting our data to the last calendar period, the HR for overall death in sepsis in individuals with CD compared with controls was 1.06 (96%CI = 0.94–1.18). When interpreting our results it is of importance to remember that this paper is a null paper and the potentially more severe disease diagnosed in the first part of our study period has not influenced our overall risk estimates more than marginally. Likewise, we abstained from examining sepsis survival in different pediatric age groups since this would lead to multiple comparisons and a risk of false positive significances.

Naturally, co-morbidities other than CD can influence the outcome in sepsis [19]. In this study we chose to adjust for type 1 diabetes mellitus, which shares several genetic traits with CD and accordingly, is more common in the CD population [33] and autoimmune thyroiditis (also associated with CD) [34]. As in celiac patients, patients with diabetes have an increased risk of acquiring sepsis compared with the general population [35]. However, studies are contradictory regarding whether this autoimmune condition affects the prognosis [36, 37]. In a study from a Taiwanese university hospital comparing 242 diabetic (both type 1 and type 2) patients with bacteraemia and 597 non-diabetic patients with bacteraemia, 30-day mortality rates were similar [37], perhaps suggesting that certain forms of underlying comorbidity are not as important for the outcome in sepsis as is rapid and correct treatment of the sepsis per se. However, an increasing number of pre-existing comorbidities have been linked to a higher sepsis mortality [36, 38].

Although this is a large study, the number of events in each subtype of sepsis was limited; in fact, for some bacteria we were unable to perform sub-analyses. The three bacterial species most strongly associated with an increased mortality in the CD group were streptococcal, pneumococcal and Gram-negative sepsis. However, because of the small numbers of cases in each subtype, we urge caution when interpreting these results. Because of the high prevalence of hyposplenism in the CD population [17] we had expected an excess mortality from pneumococcal sepsis in CD. Studies have shown that individuals with CD seem to have an increased risk for pneumococcal infections [11, 12]. Pneumococci (and Haemophilus influenzae and meningococci) are encapsulated bacteria and the spleen is an important part of the immune system’s defence against such bacteria [39]. Based on our results CD seems to influence the incidence of pneumococcal disease but not its prognosis. Likewise, data has shown that individuals with CD are more prone to acquire sepsis, but again, our results suggest that the outcome of the infection is unaffected by CD. Given the high mortality rate in sepsis, it is possible that the contribution of a concomitant CD diagnosis is too small to have a statistically detectable impact on death rates.

Several studies have evaluated the risk for infections in individuals with CD [11, 12] but to our knowledge this is the first study to focus on survival in sepsis according to CD status. The major strengths of our study are its large size, long follow-up and use of histopathology data to ascertain CD [22]. We used biopsy data from all pathology departments in Sweden to identify a representative cohort with CD patients. Biopsies showing VA was the gold standard for CD diagnosis in Sweden throughout the whole study period. Histopathology examination was carried out by a large number of pathologists (28 pathology departments between 1969 and 2008). It was not possible to carry out an agreement rate for diagnosis between all pathologists, but when representatives from the different pathology departments carried out a blinded test, 90% (95%CI = 87–94%) of biopsies with VA (according to the national steering group of small intestinal pathology) were correctly classified [22]. In most western countries, a substantial proportion of celiac patients remains undiagnosed (and are hence false-negative). However, that proportion is unlikely to constitute more than 1–2% of the control group. That 1–2% of the controls suffer from CD is however not likely to have influenced the base-line survival rate used for comparison in our study (for instance, a survival of 5 years in a false-positive control and 10 years in truly CD-negative controls would yield an average survival of 9.95 years if 1/100 controls and CD).

However the study also has some limitations. We do not have data on dietary adherence. However, since there was no statistically significant difference in risk estimates between the groups that knowledge is of less importance in this paper. Likewise we miss data on medication. Recent data suggests that that olmesartan can cause villous atrophy. Of note, olmesartan is not used in Sweden, and other ACE-inhibitors have not been linked to villous atrophy [40].

While the specificity of a sepsis diagnosis among patients in intensive care units in Sweden is extremely high (>99%) the sensitivity is lower (51%) [41]. A non-optimal sensitivity however, is unlikely to have influenced the risk estimates in our study. It is possible that the diagnostic accuracy for sepsis is better in intensive care units than in other departments, but validation studies of the Swedish Patient Register have shown that in general 85–95% of the diagnoses are correct [24]. Likewise, misclassification of sepsis is unlikely to depend on CD status.

We are not aware of any recent validation of the Swedish Cause of Death Register, but missingness is rare (1.1% in 2013) [42]. A Swedish study from 1995 showed that in a number of cases there was a discrepancy between death certificates and hospital discharge conditions indicating certification errors [43]. However, sepsis was not evaluated and it is unlikely that incorrect cause of death certificates have influenced our mortality rates more than marginally.

In conclusion, this study found an increased overall mortality in patients with sepsis and concomitant CD compared with sepsis patients without CD. Still CD did not influence survival in sepsis per se.

## Supporting Information

S1 File Comparison of small intestinal histopathology classifications. (DOCX)

S2 File International classification codes for sepsis used in this paper. (DOCX)

S3 File International classification of disease (ICD) codes for comorbidities used in this paper. (DOCX)

## Abbreviations

CD: Celiac disease
aHR: adjusted Hazard ratio
CI: Confidence interval
VA: Villous atrophy
ICD: International classification of disease
