# Supplementary material for: Does Celiac Disease Influence Survival in Sepsis? A Nationwide Longitudinal Study
Source: PLoS One. 2016 Apr 28;11(4):e0154663. doi: 10.1371/journal.pone.0154663 (PMC4849637; doi:10.1371/journal.pone.0154663)
Supplement: S1 File — (DOCX) [file pone.0154663.s001.docx]

**Supporting information file 1 (Appendix 1)**

**Comparison of small intestinal histopathology classifications**

| **Classification used in this project** | **Normal** | **Inflammation** | | **Villous atrophy** | | |
| --- | --- | --- | --- | --- | --- | --- |
| Marsh Classification* | Type 0 | Type 1 | Type 2 | Type 3a | Type 3b | Type 3c |
| Marsh  Description | Pre-infiltrative | Infiltrative | Infiltrative-hyperplastic | Flat destructive | | |
| Corazza et al (*ref A*) | - | Grade A | | Grade B1 | | Grade B2 |
| SnoMed Codes | M0010, M0011 | M40000, M41000, M42000, M43000, M47000, M47170 | | M58,  D6218,  M58005 | M58,  D6218,  M58006 | M58,  D6218,  M58007 |
| KVAST/Alexander classification | I  Normal | II  Intraepithelial lymphocytosis (IEL)# | | III  Partial VA | IV  Subtotal VA | IV  Total VA |
|  |  |  |  |  |  |  |
| *Characteristics* |  |  |  |  |  |  |
| Villous atrophy | - | - | - | + | ++ | ++ |
| IEL# | - | + | + | + | + | + |
| Crypt hyperplasia | - | - | + | + | ++ | ++ |

*We did not include Marsh type 4 in this classification since such lesions are very rare and cannot be identified through SnoMed Codes.

# Increased intraepithelial lymphocyte count (often >30/100 epithelial cells).

KVAST: Kvalitets- och Standardiseringskommittén (English: Committee for Quality and Standardisation).

Ref A: Corazza GR, Villanacci V, Zambelli C, et al. Comparison of the interobserver reproducibility with different histologic criteria used in celiac disease. Clin Gastroenterol Hepatol 2007;5:838-43.
