# Supplementary material for: Does Celiac Disease Influence Survival in Sepsis? A Nationwide Longitudinal Study
Source: PLoS One. 2016 Apr 28;11(4):e0154663. doi: 10.1371/journal.pone.0154663 (PMC4849637; doi:10.1371/journal.pone.0154663)
Supplement: S2 File — (DOCX) [file pone.0154663.s002.docx]

**Supporting information file 2 (Appendix II)**

**International classification codes for sepsis used in this paper**

|  | ICD-7 | ICD-8 | ICD-9 | ICD-10 |
| --- | --- | --- | --- | --- |
| Sepsis# | 053; 057.1 | 036.0; 036.1; 038.0-2; 038.8; 038.99 | 036C; 038 | R65.0; R65:1  A39.2; A40-41 |
|  |  |  |  |  |
| Any streptococci | 053.0 | 038.0 | 038A | A40.0; A40.1; A40.2 |
| Pneumococci | 053.2 | 038.2 | 038C | A40.3 |
| Meningococci | 057.1 | 036.0;  036.1 | 036C | A39; (A39.2 restricted) |
| *Haemophilus influenzae* |  |  |  | A41.3 |
| Gram-negative bacteria |  | 038.8† | 038E | A41.5 |
| Anaerobic bacteriae |  |  | 038D | A41.4 |
| Staphylococci § | 053.1 | 038.1 | 038B | A41.0-2 |

#We did not include sepsis after abortion, transfusion, surgery, or immunisation or while giving birth. Nor did we include sepsis that was specified as caused by actinomycosis, anthrax, candidiasis, salmonellosis, extraintestinal yersiniosis, gonococci, listeriosis, meiloidosis, yersinia pestisor tularaemia.

† In ICD-8 there are available data only on E Coli.

§ In older classifications “Staphylococci” were not divided into “Staphylococcus Aureus” and “other Staphylococci”. For that reason we looked at any Staphylococcal sepsis throughout the study period.
