# Supplementary material for: Does Celiac Disease Influence Survival in Sepsis? A Nationwide Longitudinal Study
Source: PLoS One. 2016 Apr 28;11(4):e0154663. doi: 10.1371/journal.pone.0154663 (PMC4849637; doi:10.1371/journal.pone.0154663)
Supplement: S3 File — (DOCX) [file pone.0154663.s003.docx]

**Supporting information file 3 (Appendix III)**

**International classification of disease ICD) codes for comorbidities in this study**

*Diabetes mellitus, type 1*: ICD7: 260; ICD8: 250; ICD9: 250; ICD-10: E10. Since the Swedish ICD system did not distinguish between type 1 and type 2 diabetes until ICD-10 we defined type 1 diabetes as having a diagnosis of diabetes ≤age 30 years of age.

*Autoimmune thyroid disease*: ICD-7: 252.00, 252.01, 252.02, 253.10, 253.19, 253.20, 253.29, 254.00, ICD-8: 242.00, 242.09, 244, 245.03, ICD-9: 242A, 242X, 244X, 245C, 245W, ICD-10: E03.5, E03.9, E05.0, E05.5, E05.9, E06.3, E06.5.
